# Peer review of "An Investigation on the Sound Absorption Performance of Granular Molecular Sieves under Room Temperature and Pressure"

_materials, 2020, doi:10.3390/ma13081936_

Round 1

Reviewer 1 Report

In this manuscript, the authors report on acoustical experiments on "granular molecular sieves". With no surprise, they find that the "molecular sieve" aspect of their material has no influence on the acoustical results. Only the size of the grains used for making the sample plays a role. 

The paper is correct, and there is no mention of a role of the molecular sieve. But the title and the abstract are misleading. Using the term of "molecular sieve" is questionable here. The paper is about sound absorption with samples made of grains of different mean radii. That is where the physics comes from, not from the crystalline structure. As a consequence, I think there is nothing new in the results shown here. I do not see the interest for potential readers and do not recommend publication. 

Author Response

Authors’ Response to the Reviewers’ comments

Dear editor,

Thank you and the reviewers for your valuable suggestions. We have carefully considered each comment made by the reviewers and made significant revisions in the paper.

Our responses to the reviewer’s questions and the detailed changes are listed below. We greatly appreciate your time and efforts to improve our manuscript for publication and we look forward to hearing from you soon.

Sincerely,

Bing Zhou, Jiangong Zhang, Xin Li and Bilong Liu

Comments from the Reviewer 1

In this manuscript, the authors report on acoustical experiments on "granular molecular sieves". With no surprise, they find that the "molecular sieve" aspect of their material has no influence on the acoustical results. Only the size of the grains used for making the sample plays a role. 

The paper is correct, and there is no mention of a role of the molecular sieve. But the title and the abstract are misleading. Using the term of "molecular sieve" is questionable here. The paper is about sound absorption with samples made of grains of different mean radii. That is where the physics comes from, not from the crystalline structure. As a consequence, I think there is nothing new in the results shown here. I do not see the interest for potential readers and do not recommend publication. 

Reply: Thank you very much for your comments. Granular molecular sieves have been successfully used as sound absorption materials inside the back cavities of miniature loudspeakers for cellphones, for example, in some type of iPhones, to reduce the first resonance frequency of the coupled membrane-cavity system significantly. however, the mechanism on the sound absorption has not been reported. It has been deduced that both the apertures among the grains and the pores due to the crystalline structure play a role in the sound absorption of granular molecular sieves. In the manuscript, we confirmed that the sound absorption is not from the crystalline structure under the condition of room temperature and room pressure. However, when the temperature and pressure are higher than that of room conditions, if the crystalline structure has an obvious effect on the sound absorption has not been convinced and worth to be investigated in the future.

We think the findings that the sound absorption is not from the crystalline structure under the room temperature and room pressure, together with the effective modelling of the sound absorption of the granular molecular sieves, are worth to be published. We wish the referee could change his position.

The authors would like to thank the editor and the reviewers very much for the comments. These comments are very helpful to improve the quality of this manuscript.

Reviewer 2 Report

The paper describes the results of acoustic impedance tube measurements of the sound absorption of different types of molecular sieve beads packed into a perforated container. A theoretical model is then used to predict the measured behavior.

Referee assessment

The paper is mostly interesting, however several pages have not converted to a readable PDF output. Therefore a full review of the manuscript is not possible. Below I have described the main issue as well as several other major points that need to be addressed. A thorough English language revision ideally by a native speaker or professional editing service is highly recommended.

  1. Parts of manuscript cannot be reviewed, pp. 7-9
    The section about the theoretical model and fitting with experimental data cannot be adequately reviewed since the PDF output is broken and full of errors. Pages 7 to 9 are full of "Error! Objects cannot be created from editing field codes." or "Error! Reference source not found."
    It is unclear whether this happened during manuscript preparation or the uploading process to the submission system. But authors must always double-check the final PDF output of submitted papers for errors like these, they should never reach reviewers.

    Additionally, part of the symbols in formulas 1-8 do not appear to be displayed correctly.
  2. Molecular sieve type description
    The type of molecular sieve is described by its pore diameter in Ångström. The Ångström (Å) is a unit of length commonly used at the atomic scale. It corresponds to 0.1 nm. Consequently, the repeated statements that the 3Å (or 3A) sieve has a pore diameter of 0.3 nm, etc., is redundant. If necessary, the readers can be reminded once that 1Å = 0.1 nm, but not multiple times.
  3. Molecular sieve and particles - nomenclature
    The authors work with a porous material called molecular sieve and describe the individual piece of sieve material as "particle". This nomenclature can cause confusion, since from the sieve point of view a particle would be an atom or molecule interacting with the pores.
    It is recommended to use a different term to avoid this confusion. The mm-sized, round pieces also seen in Fig.1 are typically called beads. Only when they are used in powder form with a few micrometers diameter are they called particles.
  4. Reference absorption
    The authors forgot to validate their absorption with reference measurements, especially in light of the bead container. The authors claim "A perforated cylindrical box is designed to fill the loose molecular sieve, and the perforated cylindrical box has no sound absorption." The statement that the box alone has no sound absorption must be validated by a reference measurement and shown at least once in the results. A validation of the (non-)absorption of the impedance tube and measurement setup without any absorber or box would also be useful.
  5. Packing density
    The authors demonstrate the effect of different bead sizes, but are missing a discussion of packing density. For one, how did the authors ensure optimal and similar packing density of their beads in the perforated box? Since the sound absorption at audible frequencies is dominated by the spaces between the beads and not the much smaller pores, the packing density will be the most dominant factor.
    Since the packing density for larger beads is smaller, the mean absorption is lower. However, the more or less regular arrangement of the beads is more pronounced, increasing multiple scattering effects.
    Showing that the measurements can be reproduced when emptying and filling the box multiple times would make the results more convincing and useful, i.e. a validation of the reproducibility is missing.

Author Response

Comments from the Reviewer 2

The paper describes the results of acoustic impedance tube measurements of the sound absorption of different types of molecular sieve beads packed into a perforated container. A theoretical model is then used to predict the measured behavior.

Referee assessment

The paper is mostly interesting, however several pages have not converted to a readable PDF output. Therefore a full review of the manuscript is not possible. Below I have described the main issue as well as several other major points that need to be addressed. A thorough English language revision ideally by a native speaker or professional editing service is highly recommended.

1. Parts of manuscript cannot be reviewed, pp. 7-9
The section about the theoretical model and fitting with experimental data cannot be adequately reviewed since the PDF output is broken and full of errors. Pages 7 to 9 are full of "Error! Objects cannot be created from editing field codes." or "Error! Reference source not found."
It is unclear whether this happened during manuscript preparation or the uploading process to the submission system. But authors must always double-check the final PDF output of submitted papers for errors like these, they should never reach reviewers.
Additionally, part of the symbols in formulas 1-8 do not appear to be displayed correctly.

Reply: Thank you for your comments. The PDF output has been checked and the symbols of formulas 1-8 have been fixed.

2. Molecular sieve type description
The type of molecular sieve is described by its pore diameter in Ångström. The Ångström (Å) is a unit of length commonly used at the atomic scale. It corresponds to 0.1 nm. Consequently, the repeated statements that the 3Å (or 3A) sieve has a pore diameter of 0.3 nm, etc., is redundant. If necessary, the readers can be reminded once that 1Å = 0.1 nm, but not multiple times.

Reply: Yes. The type of molecular sieve according to pore size is described, and the Ångström (Å)  is briefly stated. The revised description is: Molecular sieves with different crystal structures are usually classified by its pore diameter, such as 3Å, 4Å and 5Å, where 1Å = 0.1 nm.

3. Molecular sieve and particles - nomenclature
The authors work with a porous material called molecular sieve and describe the individual piece of sieve material as "particle". This nomenclature can cause confusion, since from the sieve point of view a particle would be an atom or molecule interacting with the pores.
It is recommended to use a different term to avoid this confusion. The mm-sized, round pieces also seen in Fig.1 are typically called beads. Only when they are used in powder form with a few micrometers diameter are they called particles.

Reply: Thank you for your comments.“Molecular sieve pellets”is used to describe this porous material in this paper.

4. Reference absorption
The authors forgot to validate their absorption with reference measurements, especially in light of the bead container. The authors claim "A perforated cylindrical box is designed to fill the loose molecular sieve, and the perforated cylindrical box has no sound absorption." The statement that the box alone has no sound absorption must be validated by a reference measurement and shown at least once in the results. A validation of the (non-)absorption of the impedance tube and measurement setup without any absorber or box would also be useful.

Reply: Yes. The sound absorption of the perforated cylindrical box alone is description and added as the lines: 115-123.

5. Packing density
The authors demonstrate the effect of different bead sizes, but are missing a discussion of packing density. For one, how did the authors ensure optimal and similar packing density of their beads in the perforated box? Since the sound absorption at audible frequencies is dominated by the spaces between the beads and not the much smaller pores, the packing density will be the most dominant factor.
Since the packing density for larger beads is smaller, the mean absorption is lower. However, the more or less regular arrangement of the beads is more pronounced, increasing multiple scattering effects.
Showing that the measurements can be reproduced when emptying and filling the box multiple times would make the results more convincing and useful, i.e. a validation of the reproducibility is missing.

Reply: Due to the coronavirus situation, we have been forbidden to enter the laboratory in the university so far. We are not able to measure the packing density and conduct the sound absorption test before the re-submission deadline. Otherwise it can be done and included in the revised manuscript. We are sorry for this deficiency.

The authors would like to thank the editor and the reviewers very much for the comments. These comments are very helpful to improve the quality of this manuscript.

Reviewer 3 Report

The authors have investigated the sound absorption characteristics of molecular sieves (zeolites) in air at standard room temperature and pressure. Effective sound transmission/ absorption through engineered materials has been an active field of research for several decades, with diverse applications in the audible and ultrasonic frequency regimes. This work presents an advancement in the current state of understanding of the sound absorption characteristics of porous media. In particular, the effects of air gap size, and layer thickness of molecular sieve assemblies is investigated. Sound absorption measurements, performed using an impedance tube apparatus, are compared with a phenomenological model. Parameters such as porosity, tortuosity, mean free path, etc. are extracted using an optimization routine. The authors further validate their model parameters through experiments on new samples with different thicknesses. 

I believe this work will be of interest to the readers of Materials, and to the acoustics research community in general. However, the manuscript, in its present form, has several deficiencies that need to be addressed before it can be considered appropriate for publication. 

  1. In the introduction section, please provide a reference in the first paragraph for the mechanism of sound absorption by porous materials. For instance, Cao, L., Fu, Q., Si, Y., Ding, B., & Yu, J. (2018). Porous materials for sound absorption. Composites Communications, 10, 25-35. or similar work could be cited here. 
  2. While defining molecular sieves in the introduction ("Molecular sieve is a kind of granular porous material and have been widely used as air adsorbent and desiccant in many applications".), please provide a reference for background on molecular sieves and their applications. For instance, Szostak, R. (1989). Molecular sieves. New York: Van Nostrand Reihold. Or cite refs [26, 27] up front here. 
  3. In the final sentence of the first paragraph of the introduction, the authors mention that there are few reports on the topic of sound absorption through molecular sieves. It is important to cite these few reports. For instance, It should be mentioned here that the underwater acoustic damping behavior using molecular sieves has been investigated previously (Philip, B., Abraham, J. K., Varadan, V. K., Natarajan, V., & Jayakumari, V. G. (2004). Passive underwater acoustic damping materials with Rho-C rubber–carbon fiber and molecular sieves. Smart materials and structures, 13(6), N99.)
  4. The description and background of molecular sieves (Lines 87 - 104) can go in the introduction instead of Section 2.1. The authors should start with the type of molecular sieves being investigated in Section 2.1.
  5. In the experimental section, please provide details of the perforated cylindrical box that was used to fill the loose molecular sieves. What is the size of the perforations in the cylindrical box? How was this chosen? Will this have an effect on the sound absorption of the molecular sieves? What was the packing density of the molecular sieve particles inside the perforations? Were they closely packed (hcp)? Please provide details.
  6. Were baseline measurements performed with no molecular sieves filled inside the perforated cylindrical box? This should prove that the cylindrical box has negligible sound absorption.
  7. Line 120 - there is a typographical error. "The pore sizes of 3A and 5A molecular sieves are 3nm and 5nm" - should be 0.3 nm and 0.5 nm.
  8. The authors mention that slight differences in some frequency bands may be caused by installation or measurement errors. In this regard, Were several measurements performed to determine repeatability of the experimental sound absorption spectra? If so, please consider adding a shaded curve to highlight the standard deviation, and a single solid curve to show the mean measurement.
  9. Since the gap between particles is very critical for sound absorption, please provide a detailed description of how the particles were packed/ filled? Was it an fcc or hcp style packing or stacking? What was the average air gap between the molecular sieve particles? Was it consistent across all eight sets of samples?
  10. Please provide brief description of how the specific acoustic resistance is computed from the acoustic absorption. How does this differ from acoustic impedance?
  11. From Section 3 onwards, there are several typographical errors relating to referencing equations/ tables and citations etc. in the draft. This makes it very difficult to follow the symbols used in the equations. Please fix.
  12. In equation 8 - the denominator term should be under square root. Please check.

After these comments have been addressed by the authors, the manuscript can be reconsidered for publication in Materials.

Author Response

  1. In the introduction section, please provide a reference in the first paragraph for the mechanism of sound absorption by porous materials. For instance, Cao, L., Fu, Q., Si, Y., Ding, B., & Yu, J. (2018). Porous materials for sound absorption. Composites Communications, 10, 25-35. or similar work could be cited here. 

Reply: Thank you for your comments. The above literature you recommended has been cited in this paper,as shown lines:23-29.

  1. While defining molecular sieves in the introduction ("Molecular sieve is a kind of granular porous material and have been widely used as air adsorbent and desiccant in many applications".), please provide a reference for background on molecular sieves and their applications. For instance, Szostak, R. (1989). Molecular sieves. New York: Van Nostrand Reihold. Or cite refs [26, 27] up front here. 

Reply: Yes. References for background on molecular sieves and their applications have been provide.

  1. In the final sentence of the first paragraph of the introduction, the authors mention that there are few reports on the topic of sound absorption through molecular sieves. It is important to cite these few reports. For instance, It should be mentioned here that the underwater acoustic damping behavior using molecular sieves has been investigated previously (Philip, B., Abraham, J. K., Varadan, V. K., Natarajan, V., & Jayakumari, V. G. (2004). Passive underwater acoustic damping materials with Rho-C rubber–carbon fiber and molecular sieves. Smart materials and structures, 13(6), N99.)

Reply: Thank you for your comments.The above reference is cited to illustrate the application of molecular sieves in underwater acoustics.

  1. The description and background of molecular sieves (Lines 87 - 104) can go in the introduction instead of Section 2.1. The authors should start with the type of molecular sieves being investigated in Section 2.1.

Reply: Yes. The description and background of molecular sieves has been described in the introduction.

  1. In the experimental section, please provide details of the perforated cylindrical box that was used to fill the loose molecular sieves. What is the size of the perforations in the cylindrical box? How was this chosen? Will this have an effect on the sound absorption of the molecular sieves? What was the packing density of the molecular sieve particles inside the perforations? Were they closely packed (hcp)? Please provide details.

ReplyYes.The perforated cylindrical box has been described in experimental part, as lines: 120-124.

Molecular sieve pellets are packed in a pressure-free natural state.Due to the coronavirus situation, we have been forbidden to enter the laboratory in the university so far. We are not able to measure the packing density and conduct the sound absorption test before the re-submission deadline. Otherwise it can be done and included in the revised manuscript. We are sorry for this deficiency.

  1. Were baseline measurements performed with no molecular sieves filled inside the perforated cylindrical box? This should prove that the cylindrical box has negligible sound absorption.

Reply: Yes.The sound absorption coefficient of the perforated cylindrical box without molecular sieves was measured and depicted in the Fig1.

  1. Line 120 - there is a typographical error. "The pore sizes of 3A and 5A molecular sieves are 3nm and 5nm" - should be 0.3 nm and 0.5 nm.

Reply:Yes. This error has been checked and fixed.

  1. The authors mention that slight differences in some frequency bands may be caused by installation or measurement errors. In this regard, Were several measurements performed to determine repeatability of the experimental sound absorption spectra? If so, please consider adding a shaded curve to highlight the standard deviation, and a single solid curve to show the mean measurement.

Reply:The measured data is the average of multiple measurements. Since the coronavirus epidemic situation prevented us from entering the laboratory to measure the data again for error analysis.We are sorry for the detail of the problem.

  1. Since the gap between particles is very critical for sound absorption, please provide a detailed description of how the particles were packed/ filled? Was it an fcc or hcp style packing or stacking? What was the average air gap between the molecular sieve particles? Was it consistent across all eight sets of samples?

Reply: Molecular sieves pellets are loosely packed in the specimen cylinder in a natural state without additional force or adhesive compaction. The sound absorption data provided in the paper were measured under the same experimental conditions.

  1. Please provide brief description of how the specific acoustic resistance is computed from the acoustic absorption. How does this differ from acoustic impedance?

ReplyThe acoustic impedance refers to the surface acoustic impedance of material, and the specific acoustic resistance is the real part of the specific surface acoustic impedance.It is explained in the experiment sectio as lines:113-120.

  1. From Section 3 onwards, there are several typographical errors relating to referencing equations/ tables and citations etc. in the draft. This makes it very difficult to follow the symbols used in the equations. Please fix.

Reply: Yes, it has been checked and fixed.

  1. In equation 8 - the denominator term should be under square root. Please check.

ReplyYes, it has been fixed.

The authors would like to thank the editor and the reviewers very much for the comments. These comments are very helpful to improve the quality of this manuscript.

Round 2

Reviewer 1 Report

I am still not convinced by the interest of this article. Is the fact that the pellets are made of "molecular sieves" really important? If yes, there should be a discussion on the effect of the manometric pore size (3 or 5Å)... My opinion is that the acoustic properties of the samples only come from the size of the pellets, which determines the micrometric pore size. Results of Table 4 go in that sense: the Λ and Λ' are found to be smaller in 5Å samples than in 3Å ones. It probably comes from the fact that 5Å samples were made from larger pellets... If the authors want to convince me that the "molecular sieve" has an effect, I need to see results with samples made of an other material (non "molecular sieve") with the same pellet size. My guess is that the absorption would be exactly the same. In the current state of their experimental results, I only see an effect of the pellet size and sample thickness.

Besides, I find the end of the abstract misleading. It is said that "Five non-acoustical parameters of molecular sieve pellets were obtained through simulated annealing. The experimental data were in good agreement with that of given by the parameter inversion method." Despite the fancy term of "simulated annealing" there is nothing more here than a fitting procedure. Applying the parameters found on 40 and 50mm-thick samples to results with samples of different thicknesses only proves that the measurements are robust. The conclusion in the core of the article is well written. But the abstract conveys the feeling that there is a model, or a numerical simulation, that can predict the values of the five JCAL parameters.

In short, I think that all the experimental results of this article are correct, and might be interesting, but to me they do not tell anything about the effect of molecular sieves. I do not recommend publication in the current form. The authors should be clearer in the abstract, title and conclusion that, as they write in their response letter "the sound absorption is not from the crystalline structure".

Author Response

Response to Reviewer 1 Comments

I am still not convinced by the interest of this article. Is the fact that the pellets are made of "molecular sieves" really important? If yes, there should be a discussion on the effect of the manometric pore size (3 or 5Å)... My opinion is that the acoustic properties of the samples only come from the size of the pellets, which determines the micrometric pore size. Results of Table 4 go in that sense: the Λ and Λ' are found to be smaller in 5Å samples than in 3Å ones. It probably comes from the fact that 5Å samples were made from larger pellets... If the authors want to convince me that the "molecular sieve" has an effect, I need to see results with samples made of an other material (non "molecular sieve") with the same pellet size. My guess is that the absorption would be exactly the same. In the current state of their experimental results, I only see an effect of the pellet size and sample thickness.

Besides, I find the end of the abstract misleading. It is said that "Five non-acoustical parameters of molecular sieve pellets were obtained through simulated annealing. The experimental data were in good agreement with that of given by the parameter inversion method." Despite the fancy term of "simulated annealing" there is nothing more here than a fitting procedure. Applying the parameters found on 40 and 50mm-thick samples to results with samples of different thicknesses only proves that the measurements are robust. The conclusion in the core of the article is well written. But the abstract conveys the feeling that there is a model, or a numerical simulation, that can predict the values of the five JCAL parameters.

In short, I think that all the experimental results of this article are correct, and might be interesting, but to me they do not tell anything about the effect of molecular sieves. I do not recommend publication in the current form. The authors should be clearer in the abstract, title and conclusion that, as they write in their response letter "the sound absorption is not from the crystalline structure".

Reply:

Yes. Thank you very much for the comments. To avoid misleading, we rewrote the title, abstract and conclusion. To make it clearer, “the sound absorption is not from the crystalline structure” has been added to the abstract and conclusion.

Reviewer 2 Report

The authors have corrected the PDF conversion mistakes and revised their manuscript to address most other issues pointed out in the initial review. The revised changes are satisfactory.

Regarding the issue of packing density, the authors should include at least a discussion of this aspect in their manuscript, even if they cannot do additional measurements. The theoretical maximum packing density can easily be calculated from the bead/pellet sizes if one considers a spherical geometry. The resulting air gaps and distances can then be compared to the wavelength/frequency of the acoustic wave, possibly supporting the theoretical model of the position of the absorption minima and maxima.

Additionally, an extensive English language proof-reading by a professional/native speaker would greatly benefit the readability of the paper.

Author Response

Response to Reviewer 2 Comments

The authors have corrected the PDF conversion mistakes and revised their manuscript to address most other issues pointed out in the initial review. The revised changes are satisfactory.

Regarding the issue of packing density, the authors should include at least a discussion of this aspect in their manuscript, even if they cannot do additional measurements. The theoretical maximum packing density can easily be calculated from the bead/pellet sizes if one considers a spherical geometry. The resulting air gaps and distances can then be compared to the wavelength/frequency of the acoustic wave, possibly supporting the theoretical model of the position of the absorption minima and maxima.

Additionally, an extensive English language proof-reading by a professional/native speaker would greatly benefit the readability of the paper.

Reply:

Thank you very much for your comments. Indeed, the packaging method is related to the porosity and therefore the sound absorption of granular materials. Additional explanation has been added to the manuscript, as lines 129-135, “It should be noted that, in the stacking state, the void ratio of bulk granular materials is described by the packing density and apparent density and is related to the sound absorption of granular materials. The void ratio is inversely proportional to the packing density of materials. Molecular sieves pellets described here are random close packing in the specimen cylinder in a natural state without additional force or adhesive compaction.” Due to the current experimental conditions, this paper did not elaborate on the packing density in detail,.We are sorry for that.

We went through the manuscript again carefully and tried to improve the readability of the paper.

Reviewer 3 Report

I thank the authors for revising the manuscript, and filling in details about experimental measurements as well as JCA model fitting/ optimization for determining inverse non-acoustical parameters and predicting the sound absorption capabilities of molecular sieves in air. 

After reviewing the revised manuscript, I would like to point out very minor typographical errors in the manuscript that need to be addressed before it is ready for publication: 

  1. On page 1 in the abstract, line 14, "The absorption coefficient of different molecular sieve pellets with different sizes, and layer thicknesses were measured through impedance tubes under the room temperature and pressure.". Change "layer thicknesses were ..." to "layer thicknesses was".
  2. On page 1 in the introduction, line 25, change "tiny pore" to "tiny pores".
  3. On page 1, line 27, fix spelling of "viscosity" (currently incorrectly spelled as "viscously").
  4. On page 1, line 39, change "Granular" to "granular" (G should not be in upper case).
  5. On page 2, line 49, change "tetrahedras" to either "tetrahera" or "tetrahedrons".
  6. On page 2, line 72, remove the word "it".
  7. On page 3, line 102, remove "in section 2". (It is already stated at the beginning of the sentence).

From a technical perspective, the authors have answered all previous questions to my satisfaction.

Author Response

Response to Reviewer 3 Comments

After reviewing the revised manuscript, I would like to point out very minor typographical errors in the manuscript that need to be addressed before it is ready for publication: 

1.On page 1 in the abstract, line 14, "The absorption coefficient of different molecular sieve pellets with different sizes, and layer thicknesses were measured through impedance tubes under the room temperature and pressure.". Change "layer thicknesses were ..." to "layer thicknesses was".

2.On page 1 in the introduction, line 25, change "tiny pore" to "tiny pores".

3.On page 1, line 27, fix spelling of "viscosity" (currently incorrectly spelled as "viscously").

4.On page 1, line 39, change "Granular" to "granular" (G should not be in upper case).

5.On page 2, line 49, change "tetrahedras" to either "tetrahera" or "tetrahedrons".

6.On page 2, line 72, remove the word "it".

7.On page 3, line 102, remove "in section 2". (It is already stated at the beginning of the sentence).

Rely:Yes.Thank you for your comments. I have made all the modifications to the above problems in paper.

Round 3

Reviewer 1 Report

With the new version of the abstract and conclusion I can now recommend publication.